# Supplementary material for: Are carbon emissions trading and green financial instruments synergistic? -Comprehensive quantitative research based on content analysis
Source: PLoS One. 2024 Mar 7;19(3):e0298601. doi: 10.1371/journal.pone.0298601 (PMC10919601; doi:10.1371/journal.pone.0298601)
Supplement: S1 Data — (ZIP) [file pone.0298601.s001.zip › Data/地级市Z_F工作报告.zip!/╒π╜¡/─■▓¿╩╨/└┤╫╘╣1⁄2╓┌║┼í╛┬φ┐╦╩2╛▌═°í┐_2017.docx]

各位代表：

　　现在，我代表宁波市人民政府向大会报告工作，请予审议，并请市政协委员和其他列席人员提出意见。

　　一、本届政府工作回顾

　　市十四届人大一次会议以来，我们在中共宁波市委的正确领导下，全面贯彻党的十八大和十八届三中、四中、五中、六中全会精神，深入学习贯彻习近平总书记系列重要讲话精神和治国理政新理念新思想新战略，统筹推进“五位一体”总体布局，协调推进“四个全面”战略布局，牢固树立五大发展理念，以“八八战略”为总纲，认真落实省委省政府各项决策部署，全面实施市委“六个加快”“双驱动四治理”战略，主动适应把握引领新常态，紧紧依靠全市人民，稳中求进、砥砺前行，较好地完成了市十四届人大历次会议确定的各项目标任务。

　　五年来，我们牢牢把握转型升级这一发展主线，综合实力进一步增强。地区生产总值达到8541.1亿元，年均增长7.6%，人均地区生产总值10.9万元，年均增长7.0%。一般公共预算收入达到1114.5亿元，年均增长11.1%。固定资产投资达到4961.4亿元，年均增长15.8%。社会消费品零售总额达到3667.6亿元，年均增长12.7%。战略性新兴产业、高新技术产业、装备制造业增加值占规上工业增加值比重分别提高到17.3%、41.2%和47.2%，三次产业比重调整为3.6：49.6：46.8。金融机构本外币存贷款余额年均分别增长9.8%和9.3%，旅游总收入年均增长13.8%，邮政业务年均增长31.9%。网络零售额年均增长62.8%，商品交易市场成交额年均增长11.8%，网上网下市场成交总额保持万亿元规模。新增境内外上市公司27家、“新三板”挂牌企业149家，直接融资年均增长54.0%。农业生态化、标准化、规模化、产业化水平稳步提升，成功创建国家现代农业示范区。宁波舟山港成为全球首个9亿吨大港，集装箱吞吐量突破2100万标箱，跃居全球第4位，集装箱海铁联运量年均增长49.9%，空港旅客年吞吐量达到779.2万人次，对外开放的海港、空港口岸全部实现国际卫生港创建。

　　五年来，我们牢牢把握创新驱动这一发展战略，新旧动能进一步转换。深化国家创新型城市建设，实施“科技领航”计划。高新技术企业从756家增加到1376家，创新型初创企业突破9000家，国家级企业技术中心达到17家。获批国家知识产权示范城市，发明专利授权量年均增长28.7%。推进“四换三名”工程，推动“制造业+互联网”融合，成为全国首批质量强市示范城市。自主培育的“甬优系列”杂交稻推广面积和亩产连创新高。人才总量突破200万人，自主申报入选国家、省“千人计划”人才分别增长4.2倍和3.8倍。高起点谋划梅山新区，扎实推进新材料科技城、国际海洋生态科技城和“千人计划”产业园等重大平台建设，中意（宁波）生态园、宁波电商经济创新园区、特色小镇等重大创新载体发展迅速，宁波杭州湾新区成为国家级产城融合示范区。全社会研发投入占地区生产总值比重从1.89%提高到2.5%，工业技改投资年均增长19.0%。

　　五年来，我们牢牢把握改革开放这一发展动力，体制活力进一步释放。以“三去一降一补”为重点，深入推进供给侧结构性改革。实施“放管服”改革，实现“四张清单一张网”全覆盖。改革商事制度，实行“五证合一、一照一码”。深化综合行政执法改革。推进农村产权制度改革，村经济合作社股份合作制改革基本完成，农村土地承包权确权登记颁证工作进展顺利。国资国企改革稳步推进，投融资体制、社会事业等领域改革持续深化。新增省级以上改革试点114个，其中中央和国家部委改革试点65个。积极建设“一带一路”倡议支点城市，港口经济圈纳入长江经济带规划和长三角城市群发展规划。连续举办浙洽会、消博会、中东欧博览会等重大展会，中国航海日论坛落户宁波，“海丝指数”在伦敦波罗的海交易所成功发布。推进贸易便利化，国际贸易“单一窗口”启用。外贸全国占比和排名稳步上升，跨境电子商务年交易额突破40亿美元。以海内外“宁波周”和甬港合作论坛为载体，深化与国内外城市合作，累计实际利用外资189亿美元、境外投资107.4亿美元，引进内资3707.4亿元，浙商甬商回归实际到位资金2776.6亿元。对口支援帮扶和“山海协作”取得新成效。

　　五年来，我们牢牢把握统筹协调这一发展方向，城乡融合和生态环境进一步提升。城市总体规划修订获批实施，推动全市域“多规融合”。中心城市能级提升，谋划建设宁波都市圈，东部新城、南部新城、镇海新城展露新姿。建成“一环六射”高速路网、“南客北货”铁路格局、“十字型”轨道交通骨架和“一纵两横”快速路网，公路、铁路里程分别增加522公里和89.4公里，打通断头路56条，实现城乡客运一体化。“提升城乡品质”行动全面实施，智慧城市建设深入推进，入选全国海绵城市建设试点，实施“百村示范千村整治”工程，建成一批示范乡镇、示范村和风景线。深化国家新型城镇化综合试点，全面取消户口性质划分，常住人口城市化率达到71.9%。创新流动人口服务管理，完成钦寸水库移民安置5080人，新老宁波人融合发展。扶持“16+3”相对薄弱地区，推动四明山区域生态发展。城乡统筹发展水平保持全国前列。打好“五水共治”“三改一拆”“四边三化”等组合拳，治理黑河臭河垃圾河1062公里，“三改”、拆违分别完成8652万平方米和7231万平方米。实施渔场修复振兴计划。整治重污染行业，淘汰黄标车11.2万辆。开展国家低碳城市试点，节能减排任务全面完成。在省内率先实施生态保护红线规划，获批建设国家生态文明先行示范区，国家生态区县（市）达到5个，森林覆盖率达到48.7%。

　　五年来，我们牢牢把握增进民生福祉这一发展目的，人民群众获得感进一步增加。财政民生支出连年加大，公共财政民生投入年均增长14%。城乡居民收入年均实际分别增长7.3%和8.2%，收入比由1.89：1缩小到1.80：1。新增城镇就业85.5万人，实现充分就业区县（市）全覆盖，城镇登记失业率保持在2.6%以下。培育创业主体53.7万户，成为全国创业先进城市。基本建成惠及全体城乡居民的社保体系，社会保障水平位居全国大中城市前列。推进被征地人员养老保障、低标准养老保险、外来务工人员社保制度向基本保险制度衔接并轨，完成居民医保城乡并轨，实施大病保险制度。户籍人员养老保障参保率从82%提高到93.6%。启动机关事业单位养老保险改革。每百名老人养老床位达到4.3张，多种形式的居家养老服务蓬勃发展。危旧房网格化监管实现全覆盖，以成片危旧住宅和城中村为重点的棚户区改造加快推进，累计改造建筑面积893.1万平方米。加强房地产市场调控，城镇住房保障覆盖率达到24.6%。义务教育均衡发展，普通高中特色多样发展，新（改扩）建中小学幼儿园800所，成为国家级职业教育与产业协同创新试验区、国际教育综合改革试验区。实施“三医联动”改革，实现国家卫生城市“四连冠”。居民健康水平得到提高，人均期望寿命达到81.24岁。深化平安宁波建设，推进公共安全、防灾减灾、应急管理工作，安全生产形势持续好转，食品药品监管得到加强，加大金融风险防范力度，有效抵御“菲特”等强台风袭击，社会保持和谐稳定。老龄、计生、工青妇、关心下一代、慈善、助残、志愿服务、红十字等事业实现新进步，民族、宗教、外事、侨务、港澳台等工作取得新成效，军民共建和国防动员工作进一步加强，实现全国双拥模范城“七连冠”。

　　五年来，我们牢牢把握增强软实力这一发展抓手，城市影响力进一步扩大。大力弘扬社会主义核心价值观，开展中国梦等主题宣传，实现全国文明城市“四连冠”。历史文化名城名镇名村保护力度加大，获得全国十大旅游休闲示范城市称号。东钱湖跻身首批国家级旅游度假区，雪窦山成为“佛教五大名山”。当选“东亚文化之都”，成为世界文化遗产城市。公共文化服务体系不断完善，基本形成“城市15分钟、农村30分钟”文化服务圈，建成农村文化礼堂893个。奥体中心加快建设，宁波健儿实现奥运金牌零的突破。文创产业增加值占地区生产总值比重提高到6.9%。哲学社会科学、档案史志得到新发展。社会管理日益精细，促进信息资源整合共享。强化政府自身建设，扎实开展党的群众路线教育实践活动、“三严三实”专题教育、“两学一做”学习教育，完善政府目标管理考核机制。推进法治政府建设，提请市人大常委会审议地方性法规草案21件，制定、修改、废止政府规章43件，办理市人大建议2830件、市政协提案2841件。强化科学民主决策，自觉接受社会、舆论等各类监督，深化政务公开，政府透明度连续位居同类城市前列，法治政府建设水平跃居主要城市首位。

　　2016年，面对诸多矛盾叠加、风险隐患交汇、经济下行压力加大的严峻挑战，在中共宁波市委的坚强领导下，我们与全市人民一道，凝心聚力、抢抓机遇，积极作为、克难奋进，各项工作再上新台阶，取得新成就，实现了“十三五”良好开局。

　　（一）全力稳增长，有效提升经济质量。在省内率先出台“降本减负35条政策”，为企业减负300多亿元。强化重大项目建设攻坚，固定资产投资增长10.1%。实施“促进外贸持续稳定增长22条意见”，自营进出口额达到6262.1亿元。培育消费新热点、新模式，社会消费品零售总额增长10.3%，网络零售额增长45.6%。实施“房地产新政16条”，库存明显减少，市场保持平稳。加大金融支持力度，保障重点领域、重点企业资金需求，统筹建立风险防控体系，有序化解“两链”风险。地区生产总值增长7.1%，一般公共预算收入增长10.5%。

　　（二）强力促转型，有效提升产业层级。强化创新驱动，获批全国首批科技成果转移转化示范区、国家小微企业创业创新基地示范城市、促进科技和金融结合试点城市，科技成果“三权”改革不断深化，宁波科技大市场、知识产权综合运用与保护第三方平台投入使用，举办全国机器人峰会，每万人发明专利授权数达到23件。强化改革推动，完善企业分类综合评价和要素差别化配置机制，设立PPP投资基金和试点项目库，成立市专项基金担保有限公司和市再担保公司，宁波股权交易中心启用，建设全国普惠金融综合示范区，获批国家“十三五”服务业综合改革试点。强化开放带动，宁波国际邮件互换局投入运营，全国服务外包示范试点城市推进建设。强化产业联动，中车宁波产业基地、华强方特东方神画等建成投用，上汽大众二期、石墨烯创新中心、中芯国际集成电路等项目加快建设，都市农业示范区、主导产业集聚区和粮食蔬菜生产功能区建设水平进一步提升，促进农旅融合，乡村旅游蓬勃发展。规上工业企业利润增长30.5%，战略性新兴产业、高新技术产业、装备制造业增加值分别增长10.4%、9.1%和11.1%，文创产业增加值增长16.5%。

　　（三）合力创平台，有效提升创新动能。推进产业平台建设，获批建设全国首个“中国制造2025”试点示范城市，智能制造加快部署推进。获批建设全国首个国家保险创新综合试验区，宁波保险创新产业园启动建设。获批建设国家跨境电子商务综合试验区，5个省级跨境电商产业集聚区发展迅速。宁波杭州湾滨海新城、航天智慧科技城、临空经济示范区开发创建加快，入选国家通用航空产业综合示范区，宁波南部滨海新区、宁南贸易物流园等开发进展顺利。推进创新平台建设，积极争创浙东南国家自主创新示范区，筹建新材料联合研究院，启动建设浙江大学宁波“五位一体”校区，推进建设宁波海洋研究院，引进共建麻省理工宁波（中国）供应链创新学院，中官路创业创新大街等“双创”平台加速打造，建成投用吉利研究院。推进重大开放平台建设，研究拟订梅山新区总体方案，积极构筑义甬舟开放大通道。推进特色平台建设，累计11个特色小镇入选省创建和培育名单，宁海智能汽车小镇成为省级示范。

　　（四）着力强品质，有效提升城乡面貌。稳妥实施新一轮行政区划调整。加快新城新区建设和城市“双修”，中山路改造提升工程基本完成，新增公共绿地面积213万平方米，“三江六岸”滨江休闲带建设扎实推进。实施城市夜景美化工程，19个城市出入口景观得到提升，宁波植物园一期建成开放，三江重要堤防基本实现防洪封闭。在省内率先出台地下空间开发利用管理办法。启动小城镇环境综合整治和治危拆违专项行动，减少地质灾害点20个，城镇危险住宅房屋解危1680幢。开展美丽乡村分类创建，农村“安居宜居美居”专项行动、垃圾“三化”处理、农业废弃物“三基本”深入实施。完善交通枢纽体系，金甬铁路、世纪大道快速路等开工建设，栎社国际机场三期等进展加快，轨道交通1号线二期等建成投用，杭甬运河实现500吨全线通航。

　　（五）倾力办实事，有效提升社会事业。城乡居民人均可支配收入分别达到51560元和28572元，分别增长7.7%和7.9%。建设农村生活污水处理设施项目502个，新增受益农户22.6万户。率先创建“污水零直排区”。全市水质优良率达到48.8%。实施全省首个设区市大气污染防治条例，市区空气质量优良天数比率升至84.7%，PM2.5平均浓度下降13.3%。新增专用停车位9.1万个，市区新建公共自行车网点239个，启动出租汽车行业改革。初步建立城乡一体居民医保制度，医用耗材采购机制改革、“云医院”建设取得成效，成为全国健康城市试点市。积极实施全面两孩政策。82.3%的外来务工人员子女入读公办学校，普惠性幼儿园招生覆盖率达到83%，宁波工程学院新校区建成投用。完善养老服务体系，入选第一批中央财政支持的居家和社区养老服务改革试点，改（扩）建养老机构24家，新增养老机构床位5004张。建立困难残疾人生活补贴和重度残疾人护理补贴制度。新建和提升农村电子商务服务点456个。入选国家首批文化消费试点城市，启动全民艺术普及工程，举办“东亚文化之都·2016宁波”活动年、中日韩青少年运动会、宁波特色文化产业博览会。推进国家食品安全示范城市和省食品安全市、区县（市）“两城联创”。十方面30项民生实事全面完成。

　　各位代表，过去五年的发展成绩来之不易。这是党中央国务院、省委省政府和市委正确领导的结果，也是全市干部群众团结奋斗、顽强拼搏的结果。在此，我代表市人民政府，向全市人民和广大建设者，向市人大代表、市政协委员，向离退休老干部，向各民主党派、工商联、人民团体和社会各界人士，向驻甬人民解放军、武警部队官兵，向在甬部省属机构，向“宁波帮”和帮宁波人士，向所有关心支持宁波发展的海内外朋友们，表示衷心的感谢！

　　我们也清醒地看到，宁波经济社会发展中还存在不少短板、矛盾和问题。主要是：转型升级步伐不够快，新旧动能转换不够有力，有效投资后劲不够足，高端人才紧缺，创新驱动尚未成为发展主动力；国际大港的综合优势没有充分发挥，城市集聚辐射带动力不够强，国际化程度不够高，研发投入不够多，与先进城市有一定差距，中心城区发展品质亟待提升，城乡和区域统筹有待加强；生态环境保护压力较大，治理效果与群众期盼尚有差距；教育、医疗、交通等公共民生服务供给还有不少短板，安全生产、食品安全、治安防控等公共安全体系仍存在薄弱环节。同时，少数政府工作人员精神不振、作风不实，一些领域腐败现象和“四风”问题时有发生，个别领导干部违纪违法造成不良影响，党风廉政建设和反腐败斗争任务依然艰巨。我们一定高度重视这些问题，采取更加有力措施，认真加以解决。

　　二、今后五年发展目标和主要任务

　　今后五年是宁波追赶跨越的五年，也是实现“振兴宁波、再创辉煌”的关键时期。大变革的时代孕育大机遇，也会有大挑战。我们要找准历史方位，以对得起宁波历史、对得起人民期望的决心，全市上下横下一条心，誓师追赶，立志超越，实现时代赋予我们这代人的新使命！

　　今后五年政府工作总体要求是：高举中国特色社会主义伟大旗帜，以邓小平理论、“三个代表”重要思想、科学发展观为指导，认真贯彻落实党的十八大和十八届历次全会精神，深入学习贯彻习近平总书记系列重要讲话精神和治国理政新理念新思想新战略，统筹推进“五位一体”总体布局和协调推进“四个全面”战略布局，坚持稳中求进工作总基调，以新发展理念为引领，以“八八战略”为总纲，认真落实省委省政府决策部署和市第十三次党代会精神，围绕早日跻身全国大城市第一方队这一总要求，追赶跨越，勇毅奋起，协同推进产业高新化、城市国际化、发展均衡化、建设品质化、生态绿色化、治理现代化，加快建设国际港口名城，努力打造东方文明之都，高水平全面建成小康社会，把宁波改革开放和现代化建设全面推向新阶段。

　　今后五年政府工作目标是：

　　发展质量更加优良。经济保持中高速增长，产业迈向中高端水平。“中国制造2025”、国家服务业综合改革等试点示范取得标志性成果，实体经济提质增效明显。战略性新兴产业、高新技术产业、装备制造业支柱地位形成，占规上工业增加值比重分别提高到22%、45%和50%以上，率先走出创新驱动、智能引领、人才支撑的转型新路子。制造业创新中心、经贸合作交流中心、港航物流服务中心地位凸显。地区生产总值、一般公共预算收入年均分别增长7.5%左右，力争在实际工作中取得更好结果。

　　平台带动更加强劲。一批重大开放平台、重大创新平台、重大产业平台活力展现，辐射带动引领作用显著增强，成为再创发展新优势、集聚发展新动能的主引擎，高端要素配置能力进一步提升，助推宁波都市圈城市极核功能大幅增强，成为亚太开放门户的强劲支撑。

　　人民生活更加殷实。就业创业更加充分，城乡居民人均可支配收入年均增长7.5%以上。建成城乡一体、更高水平的社会保障体系，基本公共服务供给质量稳步提高。优质教育资源进一步丰富，劳动年龄人口平均受教育年限增加。医疗保障水平进一步提高，居民健康水平持续提升。高水平全面建成小康社会，市民幸福感、获得感进一步增强。

　　港城品质更加彰显。打造宁波都市圈，重构城市空间格局，全面提升城市综合竞争力和区域影响力，争创国家中心城市。宁波舟山港集装箱吞吐量年均增长5%左右，现代化综合交通运输体系全面形成，创新港城联动路径，国际强港地位确立。创建新型智慧城市，城市管理精细化、网格化、智能化水平不断提升，品质城区、特色城镇和美丽乡村建设深入推进。全方位展现“东方大港、江南水乡、文献之邦、商埠名城”的独特魅力。

　　城乡文明更加提升。社会治理机制更具效率，法治宁波建设深入推进，社会诚信体系更加健全，厉行法治、崇德向善成为全社会的主流价值观。市民文明素质全面提升，全区域高水平精神文明建设取得硕果，全国文明城市和东亚文化之都影响力不断增强，成为书香之城、音乐之城、影视之城。文创产业增加值占地区生产总值比重达到9%以上。

　　生态环境更加优美。绿色发展方式和生活方式成为主流，能源和水资源消耗、建设用地、碳排放总量得到有效控制，加快构筑城乡一体的绿色生态体系，市区空气质量优良天数比率达到90%以上，水质优良率达到70%以上。美丽宁波建设全域化，城乡天更蓝、水更清、地更绿、环境更优美，成为生态宜居、山海宜游、现代宜业之城。

　　今后五年，要重点突出以下六个方面：

　　（一）聚焦产业高新化，培育更加强劲的发展动能。围绕打造一流的创新型城市，坚持把创新作为引领发展的第一动力，深化供给侧结构性改革，培育以智能经济为代表的新技术新产业新业态新模式，改造提升传统优势产业，新旧动能加速转换。

　　深化创新驱动战略。构建“一带两湾”创新空间，建设创新大平台、创新大项目、创新大团队、创新大企业、创新大环境，打造一流的创新生态链。强化企业创新主体地位，鼓励企业建设研发机构，支持产学研共建创新联盟。推动科技与金融深度融合，建立军民融合创新体系。全社会研发投入占地区生产总值比重提高到3.2%以上，每万人发明专利授权数达到38件。深化人才强市战略，深入实施“3315计划”“泛3315计划”和“技能宁波”行动计划，推进职业教育“三名工程”，大力引进国内外优秀人才，人才规模达到280万人，加快建设人才新高地。加快高等教育跨越发展，优化结构、提高质量，引进共建国内外高水平大学。加强知识产权保护，推进国家知识产权区域布局试点。

　　大力振兴实体经济。建设“中国制造2025”试点示范城市，构建“3511”先进制造业体系，以智能经济为主攻方向，打造一流的现代产业体系。修复传统动能、培育新的动力，推进“拆治归”，加大“四换三名”“互联网+”“机器人+”“标准化+”力度，打造标准强市、质量强市、品牌强市。加快农业现代化，加大农业供给侧结构性改革力度，培育农业新主体、新业态、新功能，促进优质高效、特色精品、绿色生态农业发展，建设绿色都市农业强市。弘扬“工匠精神”，培育“大港工匠”“首席工人”。鼓励企业专注主业、做强实业，壮大行业领军企业和“单项冠军”队伍。

　　促进服务业跨越发展。形成“一核两圈四带”的服务业发展布局，服务业增加值占地区生产总值比重达到50%以上。发展港航物流、节能环保、金融服务、会展贸易、科技信息等生产性服务业，促进与先进制造业互动发展、深度融合，壮大港口服务经济、临空经济和总部经济。发展现代商贸、文化创意、健康服务等生活性服务业，促进实体零售、传统商贸业创新转型，建设邻里中心、智慧商圈，推进全域旅游，推动生活性服务业向精细化和高品质转变。培育平台经济和分享经济等新业态。建设国家保险创新综合试验区，实施“保险+”发展战略。加大新一代物联网基础设施建设力度，推进“中国快递示范城市”建设，争创国家5G试点城市。

　　（二）聚焦城市国际化，推动更高水平的开放合作。围绕打造一流的开放强市，坚持开放战略不动摇，依托港口资源和开放优势，建设“一带一路”倡议支点和长江经济带龙头龙眼。

　　增强港口发展能级。支持宁波舟山港创新转型发展，统筹推进江海联运服务中心建设，加快构建海铁联运、江海联运等多式联运体系，打造现代化国际强港。完善交通大格局，构筑沿海南北、沿江东西、甬昆西南三条对外综合运输大通道。构建“两环十射四连四疏港”高速公路网，新增高速公路里程111公里。推进金甬铁路、甬舟铁路、沪嘉甬铁路、城际铁路等项目，谋划甬台温沿海高铁、沪甬跨海大通道（金山—慈溪）建设。建成投用栎社国际机场三期和宁波杭州湾、宁海通用航空机场，空港旅客年吞吐量突破1100万人次。

　　提升对外开放水平。创新外贸发展体制和政策，促进外贸优进优出。探索投资贸易便利化新模式，加快“三互大通关”建设。打造国际贸易中心城市，年自营进出口额达到7000亿元。坚持招强引优，引进更多世界500强企业、全球领先企业、国际创新型企业，力争引进外资200亿美元以上。支持有条件企业走出去，培育本土跨国公司。扩大国际交流合作，举办更多国际知名展会、高端体育赛事和文化活动。

　　做强对外合作平台。举全市之力创建国家级梅山新区。整合提升开放平台，推动海关特殊监管区域向自由贸易区转型，巩固宁波在浙江对外开放的龙头地位。推进义甬舟开放大通道建设，构建甬台舟协同发展机制。加快宁波都市圈和上海、杭州都市圈城际协同发展，进一步融入长三角城市群。

　　（三）聚焦发展均衡化，实现更高层级的统筹协调。围绕打造一流的幸福民生之城，坚持以优化资源要素配置来统筹生产力布局，促进经济社会协同、城乡区域融合、公共服务共享。

　　统筹区域空间。开展城市中长期发展战略研究，编制新一轮城市总体规划，推进“多规合一”，构筑“一核两翼、两带三湾”多节点网格化市域空间格局。推动“东集聚、南融合、西开发、北提质”，提升中心城区极核功能。推进余姚、慈溪和宁波杭州湾新区“北翼”协同发展，加快宁海、象山“南翼”统筹发展，支持卫星城和中心镇建设。常住人口城市化率达到75%。促进海港、海湾、海岛联动发展，加快国家海洋经济发展示范区建设。

　　强化城乡一体。城市基础设施向村镇延伸，加快构建城乡一体的交通路网、信息网、资源管网、防洪排涝网、污废处理网，实现联网对接、高效运转。城市教育、医疗卫生、文化、体育等优质资源向农村延伸，城乡公共资源均衡配置，努力让城乡居民都能享受高质量基本公共服务。争创城乡统筹发展示范城市。

　　促进民生幸福。实施更积极的就业政策，新增就业75万人。增加城乡居民收入，持续缩小城乡居民收入比。构筑更加公平可持续的社会保障网，实现法定人员参保全覆盖。实现城乡居家养老服务全覆盖，每百名老人养老床位达到5张以上。完善住房保障体系。促进义务教育优质均衡发展，推动职业教育创新发展，健全终身教育体系。建设健康宁波，建成覆盖城乡居民的基本医疗卫生制度。健全社会救助体系。完善计划生育服务管理，促进人口均衡优质发展。

　　（四）聚焦建设品质化，打造更具魅力的现代都市。围绕打造一流的城市品质，坚持外塑形象、内炼品质，合理布局、优化功能，协同推进宁波都市圈和宁波都市区建设，提升中心城市集聚辐射功能。

　　提升城市功能品质。构筑大都市多中心体系，做精三江口，做强东部新城、南部新城、镇海新城，规划开发两江北岸，高起点高水平优化东钱湖区域发展。推进城市设计试点，促进城市有机更新。建设地下综合管廊、海绵城市，改造成片危旧房、城中村等棚户区，整治城区主要道路和街区，塑造城市核心地标。加快推进轨道交通、“四横五纵”快速路网和大型过江设施建设，创建国家公交示范城市，构建都市区一小时交通圈。突出城市滨水特色，打造“三江六塘河”，实施防洪排涝“2020行动计划”。建设城市核心商圈，优化城乡商业布局，打造区域性国际消费中心城市。完善城市管理体制，扩大智慧城管覆盖面，推进城市管理向城市治理跨越。

　　打造美丽镇村。开展“农村环境卫生整治、农村安居宜居美居、农村生态环境建设、美丽乡村示范分类创建”四大专项行动，推进村庄设计工作。发展农村美丽经济和乡村旅游，保护利用历史文化村落和传统镇村，培育提升一批有特色、有韵味、有风情的示范县、示范乡镇、示范村和风景线。

　　创建高水平文明城市。弘扬宁波精神，挖掘浙东文化、阳明文化、“宁波帮”文化等历史文化底蕴，增强文化自觉和文化自信，打造一流的城市软实力。完善现代公共文化服务体系、优秀传统文化传承体系和历史文化名城保护体系，筹建宁波新音乐厅、文化馆、河海博物馆、非物质文化遗产展示中心等设施。支持国防建设，争创全国双拥模范城“八连冠”。实施市民文明素质提升工程、文化精品创优工程和文化名人培育工程，深化媒体融合改革，推动文化事业文化产业大发展大繁荣。

　　（五）聚焦生态绿色化，建设更加宜居的美好家园。围绕打造一流的生态文明先行示范区，坚持践行“两山”重要思想，让生态优美、环境友好成为宁波的金名片。

　　整治修复城乡环境。打好“五水共治”“五气共治”“三改一拆”“四边三化”等组合拳，建成“污水零直排区”，实现城镇截污纳管和农村生活污水治理全覆盖。保护修复山水林田湖生态，筑牢以四明山绿色生态安全涵养区、沿海蓝色生态带、三江流域休闲生态走廊为骨架的生态安全屏障。增加公园绿地，创建国家生态园林城市。

　　节约集约利用资源。深化国家低碳城市试点，实施碳排放、能源消耗、水资源消耗、建设用地等总量和强度“双控”。支持节能低碳产业和新能源、可再生能源发展。实行生活垃圾强制分类，推进建筑垃圾资源化利用。倡导绿色健康生活方式、消费模式和行为习惯。

　　建设生态文明制度。建立统一的自然资源确权登记体系，健全多元化生态补偿机制，完善用能权、碳排放权、排污权有偿交易和差别化价格机制。推进环境污染第三方治理和相关产业发展。实施最严格的环境和耕地保护制度，强化生态文明建设考核和责任追究机制。

　　（六）聚焦治理现代化，营造更加优良的发展环境。围绕打造一流的制度环境，坚持依法治市与以德治市相结合，完善政府治理与社会自我调节、居民自治良性互动机制，推进地方治理体系和治理能力现代化。

　　建设法治政府。打造一流的法治城市，坚持科学民主决策，严格依法行政，自觉接受监督。深化“最多跑一次”改革，推进行政执法体制改革，推行政务公开，打造零障碍、低成本、高效率的发展软环境。深化政府系统党风廉政建设和反腐败斗争。

　　创新治理机制。培育壮大各类社会组织，拓展人民团体、基层自治组织、企事业单位等参与社会治理渠道，创建国家社会信用体系建设示范城市。加快基层治理“四个平台”建设，完善镇乡（街道）社会服务中心和村（社区）便民服务中心功能，提高网格化管理效率，打造一流的社会治理体系。

　　确保和谐稳定。健全社会矛盾疏导和联合调处机制，防范管控化解各类社会风险，引导群众依法行使权利、表达诉求、解决纠纷。加强国家安全工作，建设平安宁波，健全社会治安防控体系，强化安全生产和食品药品安全监管，努力让城市更加安全、社会更加安定、百姓更加安宁。

　　三、2017年政府主要工作

　　2017年是实施“十三五”规划承上启下的重要一年，是供给侧结构性改革的深化之年，也是新一届政府的开局之年。做好2017年政府工作，意义十分重大。综合考虑各种因素，2017年我市经济社会发展主要预期目标为：地区生产总值增长7.5%左右，一般公共预算收入增长7.5%，固定资产投资增长10%，社会消费品零售总额增长9%，外贸进出口回稳向好，城乡居民收入与经济增长保持基本同步，新增城镇就业15万人，城镇登记失业率3.6%以内，居民消费价格涨幅3%左右，节能减排降碳完成考核目标。做好2017年工作，必须坚持稳中求进工作总基调，牢固树立和贯彻落实新发展理念，主动适应把握引领经济新常态，贯彻落实全国、省两会，中央、省市经济工作会议和市第十三次党代会精神，以推进供给侧结构性改革为主线，加强预期引导，深化创新驱动，全面做好稳增长、促改革、调结构、惠民生、防风险各项工作，尤其要集中精力、善用财力、精准发力，在重大项目建设、重要试点示范、重点区块开发、重要改革创新等领域上奋力突破。重点抓好以下十方面的工作：

　　（一）狠抓实体实业，在振兴实体经济上奋力突破。实体兴，则宁波兴。按照中央、省委对宁波、杭州“唱好双城记”要求，进一步树立“大抓实体企业、大兴民营经济”的鲜明导向。强力推进“中国制造2025”试点示范城市建设，把智能制造作为主攻方向。市财政三年内安排100亿元以上资金进行精准扶持，实施智能制造、绿色制造、单项冠军培育等八大重点工程，抓好石墨烯应用示范、中芯国际集成电路等项目，全年规上工业增加值增长7.5%以上。滚动实施小微企业三年成长计划，扶持一二三产各类中小企业，培育创新型初创企业，壮大高新技术企业和创新型领军企业，充分发挥标杆企业的龙头示范作用。全力为企业降本降负，让市场主体有切身感受。落实研发投入加计抵扣等税收优惠政策。推动与部门审批事项相关的中介市场开放，让市场主体自主选择中介机构。推行工业用地弹性出让、允许工业用地分割转让等举措，继续落实企业减负政策，降低企业生产经营成本特别是制度性交易成本。全力为企业加油加劲，促进资源要素向实业型企业倾斜。推行差别化供地，建立企业综合要素分类评价机制。

　　支持企业境内外上市和“新三板”挂牌，发挥宁波股权交易中心作用。筹建金融租赁公司、地方金融资产管理公司等，完善政银担风险分担机制，加强对实体经济的金融支持。全力为企业助阵助威，加大创新激励、企业走出去等支持力度。扩大内外销产品“同线同标同质”实施范围，鼓励优先使用本土企业优质产品，推进创新产品首购首用和推广应用，建立健全促进放心消费机制，打造放心品牌，落实知识产权保护等政策，激发企业家创业创新热情。

　　（二）狠抓创新驱动，在加快动能转换上奋力突破。打造“一带两湾”创新空间，推进新材料科技城、国际海洋生态科技城和航天智慧科技城、中官路创业创新大街等建设，争创以宁波国家高新区为核心的浙东南国家自主创新示范区。推进多种形式高等教育发展，建设中国科学院大学材料学院、浙江大学宁波“五位一体”校区、宁波大学梅山校区等。推进企业创新能力建设，支持宁波新材料联合研究院、宁波智能制造产业研究院、万华新材料研究院、智能制造（气动）产业园等发展。启动宁波“创新2025”重大专项，完善科技大市场运行机制，建设国家科技成果转移转化示范区，深化国家职业教育与产业协同创新试验区发展，实施产学研协同创新。建设国家保险创新综合试验区，支持保险产品创新，推进保险创新产业园、中国保险博物馆建设，举办中国保险创新发展论坛，力争新增保险法人机构2家。抓好国家促进科技和金融结合试点、通用航空产业综合示范区、中交城投城市转型示范区等试点示范，推进产业集聚区、开发园区创新发展，新创建和培育30个省市县特色小镇，提升发展众创空间。全社会研发投入占地区生产总值比重达到2.65%。

　　（三）狠抓项目支撑，在扩大有效投资上奋力突破。抓投资就是抓发展、抓转型、抓后劲。紧盯新兴产业、基础设施、节能环保、社会民生等重点领域，充实项目储备库，动态编制三年滚动项目计划。完善招商引资体系，增强政策统筹力度，开展精准招商攻坚战，掀起项目建设大热潮，确保浙商甬商回归资金总额484亿元，实际利用外资35亿美元。加强产业项目投资，加快启动宁波石化区重大项目建设、大榭石化产品升级等项目前期，建设绿色石化基地，开工建设吉利新能源汽车、强基精密制造园一期等项目，推进建设比亚迪新能源汽车、知豆纯电动车、吉利DMA整车、集成电路特色产业园、浙东云数据产业园等项目。加强基础设施投资，开工建设葛岙水库、姚江上游“西分”等防洪排涝工程，启动东海涂防浪堤、宁海抽水蓄能电站、慈溪杭州湾联网供水等项目，加快建设各类交通基础设施。加强民生领域投资，抓好奥体中心、华强公园二期、华侨城欢乐海岸等项目推进，启动建设杭州湾湿地公园二期、宁波植物园二期，加快宁波市第一医院异地建设前期工作进程。全年重大产业项目投资、重大基础设施投资、重点技术改造投资和生态环保投资均增长15%以上。提振民间投资信心，降低市场准入门槛，完善特许经营流程，推进一批PPP项目，力争民间投资增长5%以上。

　　（四）狠抓改革攻坚，在增强发展活力上奋力突破。深化供给侧结构性改革，加大“三去一降一补”力度。淘汰落后产能，优化产能结构。实施差别化要素价格，建立统一的节能节地节水、环境、技术、质量、安全等市场准入标准。开展国家土地二级市场试点，建立统一的二级市场交易平台。推进财税、投融资等领域改革，建立完善政府性债务风险预警和应急处置机制，组织开展债务风险评估和预警。加大国有资产整合重组力度，健全国有企业法人治理结构。对接国家投资项目在线审批监管平台和公共资源交易平台，深化市县两级审批层级一体化，构建基本建设项目、商事登记、民生事务等方面的快速审批体系。启动第一批行业协会商会与行政机关脱钩试点。支持部队军改工作。深入实施国家“十三五”服务业综合改革试点、国家新型城镇化综合试点、国家文化消费城市试点。

　　（五）狠抓开放合作，在提升国际化水平上奋力突破。做大做强重点开放平台，全力建设梅山新区，促进宁波保税区、梅山保税港区等创新发展，加快中意（宁波）生态园、中捷产业园、象保合作区、大榭穿鼻岛低碳能源国际贸易中心等建设。实施参建“一带一路”经贸合作行动计划，办好海内外“宁波周”活动和浙洽会、消博会、中东欧博览会、中国航海日论坛、中国智博会、中国机器人峰会等重大展会。建设海上丝路航运大数据中心，加快海铁联运、江海联运发展，集装箱海铁联运量增长20%以上。优化外贸结构，扩大自主品牌产品和服务贸易出口，力争服务贸易总额增长10%左右。实施新一轮外贸实效企业培育工程，推进口岸诚信企业建设。争创中东欧国家投资贸易综合试验区，建设中东欧国家贸易便利化国检试验区，提升国家进口贸易促进创新示范区建设水平，推进汽车平行进口试点。建设国家跨境电子商务综合试验区，谋划布局公共海外仓、服务平台，规划建设国际邮件互换局二期，争创全国网络市场监管与服务示范区，跨境电商交易额力争突破80亿美元。支持企业走出去，建设中东欧（宁波）产业园等境外产业园，境外中方核准投资额增长10%。深化国内区域合作，促进都市圈城际协同发展，完善甬台舟协同发展机制，支持宁波杭州湾新区、余慈地区建设沪甬合作示范区。推进对口支援帮扶和“山海协作”。

　　（六）狠抓品质提升，在完善城市功能上奋力突破。启动新一轮城市总规编制前期工作，推进“东集聚、南融合、西开发、北提质”。完善落实行政区划调整配套政策，优化统筹发展机制，增强中心城区极核功能。加强城市管理标准体系建设。推进棚户区改造和城市街道、立面、道路改造，推广绿色建材，发展装配式建筑，加强物业管理行业指导。提高东钱湖区域建设水平，创建天一阁·月湖国家5A级景区，加快主城区主干道及两侧综合提升，建成三江口公园、滨江大道及景观提升工程。加强交通拥堵治理，调整完善公交管理体制，优化停车设施布局。推进栎社国际机场三期、梅山港区6号至10号集装箱码头等空港海港项目。建设金甬铁路，推进沪嘉甬铁路、甬舟铁路前期。加快杭甬高速复线、穿山疏港高速梅山连接线、象山湾疏港高速、三门湾大桥及接线、石浦高速连接线建设，建成杭州湾跨海大桥杭甬高速连接线。续建或开建北外环东段、机场路南延、世纪大道、胜陆公路等快速路。建设中兴大桥、三官堂大桥、邵家渡大桥等。推进轨道交通2号线二期、3号线一期、4号线、5号线一期和宁波至奉化城际铁路工程，开通宁波至余姚城际列车。

　　（七）狠抓强农惠农，在“三农”转型发展上奋力突破。深化农业供给侧结构性改革，健全以优质安全绿色为导向的农业政策扶持体系。贯彻大粮食安全观，加强粮食安全责任制考核，保障“菜篮子”产品生产供应。推进国家现代农业示范区建设，发展绿色都市农业，做强农业特色产业，创建省级农业产业集聚区1个、省级特色农业强镇4个，新增现代农业庄园20个。发展乡村旅游，推动农旅产业融合，提升农产品加工业，加快农村一二三产融合发展。推进平原绿化和森林建设，实施渔场修复振兴暨“一打三整治”专项行动。培育种养大户、家庭农场、专业合作社、农业龙头企业等现代农业经济主体，新增农民专业合作社35个、市级示范性家庭农场30个、农业龙头企业15家。强化金融支农，扩大农业保险覆盖面。加大农村公共服务财政投入，保障村级日常运行经费。加快相对薄弱区域发展，加大精准扶贫力度，促进低收入农户增收10%以上。推进农村集体产权制度改革，加快生产、供销、信用“三位一体”农民合作经济组织体系建设，深化农村股份合作制改革。稳妥推进“房地一体”农村宅基地及住房确权登记发证工作，基本完成农村土地承包经营权确权登记颁证，开展农村承包土地经营权抵押贷款试点。

　　（八）狠抓环境治理，在建设美丽宁波上奋力突破。强力推进“五水共治”，提标改造扩建城镇污水处理厂，进一步落实“河长制”，加快“污水零直排区”创建。奋战200天，坚决剿灭劣Ⅴ类水。推动海绵城市全域化建设。强力推进“五气共治”，加快镇海电厂搬迁，PM2.5年均浓度控制在43微克/立方米以下。加快电动汽车充电基础设施和城乡家庭屋顶光伏工程建设。治理农业面源污染，开展废弃矿山土地资源再生行动。强力推进“三改一拆”，完成“三改”1200万平方米、拆违1400万平方米。抓好小城镇环境综合整治，首批创建的40个镇乡（街道）通过省级考核验收，整治项目进度超过30%。以景区的理念和标准打造美丽乡村，开展分类创建，试点乡村规划师制度，推进农村“安居宜居美居”专项行动，深化农村环境综合整治。建设美丽乡村合格村200个以上、示范村30个、示范乡镇和风景线各15个（条）以上。加强四明山区域、象山港区域等重点生态功能区保护和利用。

　　（九）狠抓文化建设，在展示文明魅力上奋力突破。弘扬社会主义核心价值观，构建文明城市全面创建、全民创建、全域创建、全城创建新格局，争创新一轮全国文明城市。加强政务诚信、商务诚信、社会诚信和司法公信建设，健全守信激励和失信惩戒机制。贯彻落实文明行为促进条例，增强“爱心宁波·尚德甬城”品牌影响力，进一步提升市民素质和社会文明程度。加快市图书馆新馆等设施建设，支持发展基层文化活动组织。实施“一人一艺”全民艺术普及工程，提升全民阅读水平，抓好“万场电影千场戏”“你选我买”阅读定制服务等文化惠民项目。加快雪窦名山建设，扩大佛教五大名山在海内外的知名度和影响力。抓好优秀传统文化传承与名城名镇名村保护利用，加大文化礼堂建设力度。建设索菲亚中国文化交流中心，办好第十五届亚洲艺术节、宁波国际马拉松赛、世界房车锦标赛等。促进文化与制造、金融、旅游、体育等融合发展，加快宁波文创港、音乐港、影视基地、广告产业园等重大产业平台建设，创建国家文化与金融合作示范区，文创产业增加值增长13%。

　　（十）狠抓惠民利民，在促进稳定和谐上奋力突破。拓宽就业和增收渠道，开展创业型区县（市）创建活动。推进普通高中特色多样发展，加快义务教育标准化学校建设，多措并举发展学前教育，提高公办幼儿园比例。制定实施“健康宁波2030”行动纲要，争创全国健康城市示范市。开展全民健身运动，探索医保余额进入体育消费新模式。深化综合医改，推进“五医联动”。实施“双下沉、两提升”，以医联体和家庭医生签约服务为抓手，建设分级诊疗制度。推进“互联网+”健康服务，鼓励引导社会办医。深化居家和社区养老服务改革、长期护理保险制度试点改革，建设医养结合型养老机构，养老机构医疗卫生服务覆盖率达到80%。推动社保从制度全覆盖向人群全覆盖转变，低保对象向因病致贫等困难家庭延伸。加强留守儿童关爱保护和困境儿童保障。完成村（居）委会换届选举，提升基层治理能力。实施新型居住证制度和流动人口量化积分。精准调控房地产市场，完善住房保障政策措施。加强对金融特别是互联网金融的监管和执法，坚决打击逃废债行为，推进清理整顿交易场所“回头看”工作。健全安全生产责任体系、重大安全风险动态分级管控机制，推进主要风险隐患专项治理“三年行动计划”、危险化学品安全综合治理，强化港区、石化区等重点区域监管，加强消防、交通、危旧房等风险隐患排查。开展地质灾害隐患综合治理专项行动。建设立体化、信息化社会治安防控体系，严厉打击违法犯罪活动。创新社会矛盾预防化解机制。完善应急管理体系，妥善应对突发事件和重大气象灾害，继续完善巨灾保险。实施最严格的食品药品安全质量追溯制度，创建国家食品安全城市，抓好国家农产品质量安全县（市）创建，让老百姓吃得安全、吃得放心。

　　我们将按照“群众提、大家定、政府办”的理念，继续办好十方面民生实事。

　　1、大气治理方面。完成剩余4台60万千瓦及以上燃煤火电机组的烟气超低排放技术改造；治理挥发性有机物排放企业100家。淘汰改造10蒸吨/时及以下高污染燃料锅炉700台以上。

　　2、环境改善方面。建设城镇污水配套管网200公里，开工建设地下综合管廊10公里以上，启动中心城区海绵城市建设项目50个。30个镇乡（街道）建成“污水零直排区”；东钱湖旅游度假区完成沿湖村庄的截污纳管工作并淘汰农用挂桨机船舶，对未达标的旅游船舶进行改造或淘汰；余姚市推进城区截污纳管工作，年度完成60%工程量。全面消除劣Ⅴ类水。完成所有城镇污水处理设施一级A排放达标改造。宁波市南区污水处理厂三期工程试运行，新增生活污水日处理能力8万吨。城区生活垃圾分类收集覆盖面达到80%，分类实施范围内公众垃圾分类知晓率达到85%。

　　3、交通出行方面。新增专用停车位4万个，其中主城区不少于2万个；新增老旧小区停车位2000个。新增（更新）新能源公交车辆500辆；市区新辟微循环公交线路6条，全市新建8个公交场站；市区新增公共自行车网点200个，新投放自行车5000辆。

　　4、就业创业方面。扶持大学生创业实体1万家，新增大学生创业3000名；新增残疾人就业1000名。

　　5、社会保障方面。户籍人员各类养老保险（障）参保率达到94%以上。完成危旧房改造100万平方米、城中村改造100万平方米，货币化安置及新开工安置房2万套。

　　6、教育惠民方面。改造义务教育基本办学条件薄弱学校20所；公办中小学校学生宿舍全部安装独立式感烟报警器；新（改）建幼儿园40所。

　　7、医疗卫生方面。深化家庭医生签约服务，签约年度内以老年人、慢性病人、孕产妇、0-6岁儿童、残疾人为重点，重点人群签约服务率达到50%以上。实施适龄妇女免费“两癌”检查。

　　8、文体事业方面。新增农村文化礼堂100个。建设村级（社区）全民健身广场、体育休闲公园等综合性全民健身场所20个，开展全民健身体育技能公益培训1万人次。

　　9、安全保障方面。食品安全抽样检验年抽检达到47000批次，样本量达到全市常住人口每千人6批次。创建放心农贸市场45个。基本消除地质灾害隐患点80处，脱险解危群众800户。新建、改扩建避灾安置点100个。完成四明山区域下山移民800户。完成红十字公益性应急救护培训20万人次。

　　10、老年服务方面。建设镇乡（街道）区域性居家养老服务中心20个以上，推进农村社区居家养老服务全覆盖。开设智能手机知识普及培训班200期，培训中老年人1万名；组建30所老年教育学院（学校）。

　　各位代表！民生民愿重千钧。民生实事项目是市政府对全市人民的庄严承诺。我们一定全力以赴把好事办好、实事办实，用实实在在成绩取信于民、造福于民，让市民生活更加幸福美好！

　　四、全面加强政府自身建设

　　各位代表！落实市第十三次党代会战略部署，完成本届政府各项目标任务，对政府自身建设提出了更高要求。我们必须牢固树立政治意识、大局意识、核心意识、看齐意识，不忘初心、勇担使命，努力建设人民满意政府。

　　坚持为民执政。全心全意服务人民群众，切实做到对人民负责、受人民监督、让人民满意。坚持深入基层、根植大众、服务人民，履行好人民公仆职责。进一步关注民生疾苦，切实办好民生实事，让民生幸福的底色更厚重，让共享发展的主题更温暖，让人民群众有更多的获得感和满足感。

　　坚持依法行政。增强法治意识和法治思维，提高学法用法能力，把法治理念贯穿政府工作全过程。推进重点领域政府立法，提高公众参与度。健全重大行政决策法定程序，加强新型智库建设，发挥政府法律顾问制度作用，做到依法决策、科学决策、民主决策。自觉接受人大依法监督、政协民主监督和监察监督、司法监督，加强政府内部层级监督和审计监督，主动接受社会监督和舆论监督。深化行政执法体制改革，做好重大行政执法决定法制审核国家试点工作，坚持严格规范公正文明执法。推进行政复议改革，做好行政复议和行政应诉工作。加强政务诚信建设，提升政府透明度，及时回应社会关切，形成良性互动。

　　坚持高效施政。以“最多跑一次”改革倒逼简政放权、优化服务，推行“双随机一公开”，推进行政审批服务窗口、服务平台、审批标准和网络数据集成改革。强化部门协同，清理隐性审批和变相审批，能简则简、应放尽放、该改即改，努力实现“一窗受理、一城通办、一体审批、一网流转”。构筑“互联网+政务服务”市域一体化审批服务体系，用政府权力的减法换取市场活力的加法，当好服务企业和人民群众的“店小二”，构建亲清政商关系，打造审批事项最少、办事效率最高、政务环境最优城市。

　　坚持实干勤政。弘扬“大干快干、苦干实干、会干巧干”精神，让改革者轻装上阵、担当者脱颖而出、实干者大显身手。狠抓工作落实，建立健全责任明晰的重点工作清单机制、奖勤罚懒的考核激励机制、常态长效的追责问责机制，严厉整肃庸政懒政怠政，坚决整治不作为、慢作为、乱作为。提升公务员队伍能力素质，实施正向激励引导，探索容错免责机制，培养勇于冲锋的“狮子型”干部、勤勉敬业的“老黄牛”型干部、勇争一流的铁军排头兵，营造撸起袖子加油干、扑下身子抓落实的良好氛围。

　　坚持从严治政。把纪律和规矩挺在前面，认真落实全面从严治党要求，把党风廉政建设和反腐败工作不断引向深入。坚决贯彻中央八项规定精神，坚持不懈纠正“四风”，努力打造忠诚干事政治过硬、担当干事本领过硬、奋发干事实绩过硬、为民干事责任过硬、干净干事廉洁过硬的宁波铁军。认真贯彻民主集中制，完善市政府工作规则。加强对重点领域、关键环节的监管，实现公共资金、国有资产、国有资源、领导干部经济责任审计全覆盖，进一步深化机制制度建设，严肃查处各类违纪违法案件。深化基层廉洁工程建设，健全小微权力清单制度，严肃查处侵害群众利益的不正之风和腐败问题。规范财政资金分配使用管理，严控“三公”消费，坚守节用裕民的正道，用政府的“紧日子”换取群众和企业的“好日子”。

　　各位代表！新的历史方位，赋予我们这代人新的历史担当。让我们更加紧密地团结在以习近平同志为核心的党中央周围，在中共宁波市委的坚强领导下，勇立潮头，勇争一流，以优异成绩迎接党的十九大和省第十四次党代会胜利召开，为早日跻身全国大城市第一方队，加快建设国际港口名城、努力打造东方文明之都而不懈奋斗！
